# Notes and News

**Published:** 1905-07-29

**Authors:** 


### Notes and News.

Mr. Philip Vaughan has promised to subscribe £5,000 to the £50,000 the committee are raising for the Bristol Royal Infirmary.

The various synagogues of London have contributed £1,161 to the Hospital Sunday Fund Collection, the total of which now exceeds £61,000.

At a special meeting the Wandsworth guardians expressed their intention of resigning in a body if the Local Government Board persisted in requiring them to reinstate one of the vaccination officers whom they have suspended.

The Finance Committee of the County Council have advised that a provisional scheme to establish a new ambulance service for London at an annual cost of £9,600 should be postponed until the Council has been recouped for the expenditure on the Holborn and Strand improvements, and know the result of the steamboat service.

The family of the late Dr. Gilbert Smith have erected a memorial to him on the road between Exeter and Lyme Regis, on the spot where last year he died of heart disease. The memorial is a massive granite seat of semi-circular form. In the back is fixed a bronze medallion portrait of the deceased physician, with a simple inscription attached. The seat will form a welcome rest to the wayfarer and at the same time effectively recall him to memory.

The delegates attending the Public Health Congress have been very busy during the past week, visiting various points of interest, and attended a special service at St. Paul's Cathedral on Sunday. The Sectional Meetings have had an immense number of interesting topics before them. The prevention of senility, a subject introduced by Sir James Crichton Browne, attracted much attention. He suggested that the natural life of man was 100 years. Child study, sanatoria, the housing question, and the London drinking water occupied attention during one day's sitting, and diet and disease, physical training, and public health dangers that of another.

The Special Appeal Committee of the Royal Waterloo Hospital for Children and Women make an urgent appeal for funds towards the new hospital; £20,000, of the sum of £50,000 required, has been received.

Through the will of the late Miss Marianne Hasker, of St. Leonards-on-Sea, King's College Hospital Removal Fund will benefit to the extent of £20,000. One of the new blocks or wings will be named after the benefactress.

The bacteriological examination of the ice cream sold in Birmingham, after partaking of which so many persons were ill, reveals that the material contained an active poison of a character to cause illness. The analysis will not, however, be published for some time.

For more than a year the Royal Society has been conducting an inquiry into the causation and history of Malta fever. A great deal of information has already been gained, and this tends to show that there is a prospect that the fever may be successfully grappled with before long.

At the annual trade dinner of the Medico-Psychological Association Dr. Coupland remarked that the amendment of the Lunacy Laws suffered neglect at the hands of the Government, and he advocated persistency until the subject received attention. He paid a high compliment to the efficiency of asylum administration in this country.

Vienna has lost a distinguished medical member of its community through the death of Professor Hermann Nothnagel, who died last week. He studied in Berlin under Traube, and worked with Virchow and Leyden. He served as an army doctor in the Franco-Prussian war, and settled finally at Vienna, where he became distinguished mainly through his discoveries in regard to the action of the heart.

We are promised before long a variety of sugar which can be safely taken by diabetics, which will compete with glucose commercially. This substance is lævulose, which has hitherto been too expensive for general use. Lævulose can be prepared from inulin, which is contained in chickory roots and dahlia tubers to a large extent. A patent has been granted to Mr. S. Stein, of Liverpool, in connection with a process to produce lævulose for commercial purposes.

The fourth Centenary Celebration of the Royal College of Surgeons of Edinburgh was held on the 20th. A large and brilliant gathering assembled for divine service in St. Giles' Cathedral, after which the Corporation of Edinburgh entertained the College at luncheon. The President, Sir Patrick Heron Watson, delivered an address of an historical and retrospective character in the McEwan Hall, after which honorary fellowships were bestowed on a large number of foreign and British medical and scientific celebrities. The kindred Colleges of England and Ireland and other societies presented congratulatory addresses, and in the evening a reception was held at the Royal Scottish Museum.

